# Identification of SNP barcode biomarkers for genes associated with facial emotion perception using particle swarm optimization algorithm

**DOI:** 10.1186/1744-859X-13-15

**Published:** 2014-05-21

**Authors:** Li-Yeh Chuang, Hsien-Yuan Lane, Yu-Da Lin, Ming-Teng Lin, Cheng-Hong Yang, Hsueh-Wei Chang

**Affiliations:** 1 Department of Chemical Engineering & Institute of Biotechnology and Chemical Engineering, I-Shou University, Kaohsiung 84001, Taiwan; 2 Institute of Clinical Medical Science, China Medical University, Taichung 40402, Taiwan; 3 Department of Psychiatry, China Medical University Hospital, Taichung 40402, Taiwan; 4 Department of Electronic Engineering, National Kaohsiung University of Applied Sciences, Kaohsiung 80778, Taiwan; 5 Department of Psychiatry, Taipei Veterans General Hospital, Hsinchu Branch, Hsinchu 31064, Taiwan; 6 Cancer Center, Translational Research Center, Kaohsiung Medical University Hospital, Kaohsiung Medical University, Kaohsiung 80708, Taiwan; 7 Institute of Medical Science and Technology, National Sun Yat-sen University, Kaohsiung 80424, Taiwan; 8 Department of Biomedical Science and Environmental Biology, Kaohsiung Medical University, Kaohsiung 80708, Taiwan

**Keywords:** Particle swarm optimization, Single-nucleotide polymorphism, SNP interaction, Facial emotion perception, Algorithm

## Abstract

**Background:**

Facial emotion perception (FEP) can affect social function. We previously reported that parts of five tested single-nucleotide polymorphisms (SNPs) in the MET and AKT1 genes may individually affect FEP performance. However, the effects of SNP-SNP interactions on FEP performance remain unclear.

**Methods:**

This study compared patients with high and low FEP performances (*n* = 89 and 93, respectively). A particle swarm optimization (PSO) algorithm was used to identify the best SNP barcodes (i.e., the SNP combinations and genotypes that revealed the largest differences between the high and low FEP groups).

**Results:**

The analyses of individual SNPs showed no significant differences between the high and low FEP groups. However, comparisons of multiple SNP-SNP interactions involving different combinations of two to five SNPs showed that the best PSO-generated SNP barcodes were significantly associated with high FEP score. The analyses of the joint effects of the best SNP barcodes for two to five interacting SNPs also showed that the best SNP barcodes had significantly higher odds ratios (2.119 to 3.138; *P* < 0.05) compared to other SNP barcodes. In conclusion, the proposed PSO algorithm effectively identifies the best SNP barcodes that have the strongest associations with FEP performance.

**Conclusions:**

This study also proposes a computational methodology for analyzing complex SNP-SNP interactions in social cognition domains such as recognition of facial emotion.

## Introduction

Facial emotion perception (FEP) is a major indicator of social function [1-3]. In schizophrenia, FEP tends to be deficient [4], and improvements in FEP are associated with improvements in occupational functioning and independent-living capability [5]. However, potential genetic associations with FEP performance have not been addressed.

Recent reports show that the Met proto-oncogene (hepatocyte growth factor receptor) (MET) and v-akt murine thymoma viral oncogene homolog 1 (AKT1) genes have important roles in psychiatric conditions [6-8]. For example, four single-nucleotide polymorphisms (SNPs) (rs2237717, rs41735, rs42336, and rs1858830) of the MET gene and the rs1130233 SNP of the AKT1 gene have been associated with schizophrenia [9-11] and with neurocognitive performance [9,11]. Our previous study [12] was the first to explore genetic factors in the associations of these five SNPs with FEP performance. Although the roles of individual SNPs have been investigated in FEP performance, the roles of SNP-SNP interactions remain unclear.

Accumulating evidence obtained by polygenetic models reveal the joint effects of multiple SNPs in various diseases [13-21]. Computational algorithm tools have improved the identification of functional SNPs [22], but not for the SNP-SNP interaction issue. Recently, various computational algorithms have been developed to investigate SNP-SNP interactions in numerous association studies [21,23-33].

This study applied the particle swarm optimization (PSO) algorithm [34] to evaluate the use of specific combinational SNP patterns and their corresponding genotypes, i.e., SNP barcodes, for discriminating between high and low FEP performances in terms of SNP-SNP interactions. The FEP performance of the PSO-generated SNP barcodes were further assessed by odds ratio (OR) analyses. In each of these two related genes, five SNP combinations were systematically assessed in terms of their potential joint effects on FEP performance.

## Methods

The SNP barcodes, i.e., the unique patterns of SNP combinations with their corresponding genotypes, were generated by a PSO algorithm [34]. The PSO is characterized by its rapid convergence, which enables rapid identification of optimal solutions in a wide solution space. Here, PSO was used for rapid identification of the optimal SNP barcodes, namely, the best SNP barcodes, i.e., those that revealed the largest differences between the high and low FEP groups.

### Introduction to particle swarm optimization

The robustness of PSO [34] results from its use of swarm intelligence to search for the optimal solution to a complex problem. The swarm intelligence can be described as a system that automatically evolves by simulating the social behavior of organisms, e.g., the social behavior of knowledge sharing. By sharing valuable information, the behaviors of individuals in a swarm are optimized to achieve a certain objective. In PSO, an individual is considered a particle, which is a vector in the problem space. The information for the particle includes knowledge gained from its previous experience and knowledge gained from the swarm. The value of the particle, which is estimated by the objective function, is used to update its information and to optimize the objective of the swarm. Therefore, the swarm can converge to exploit good resolution in local regions of the problem space; the common objective can also be updated when one particle finds a better objective so that the particle can lead the swarm in exploring a different region of the problem space. These superior search characteristics have made PSO the most popular evolutionary algorithm in several fields.

### PSO definition

**Figure 1:**
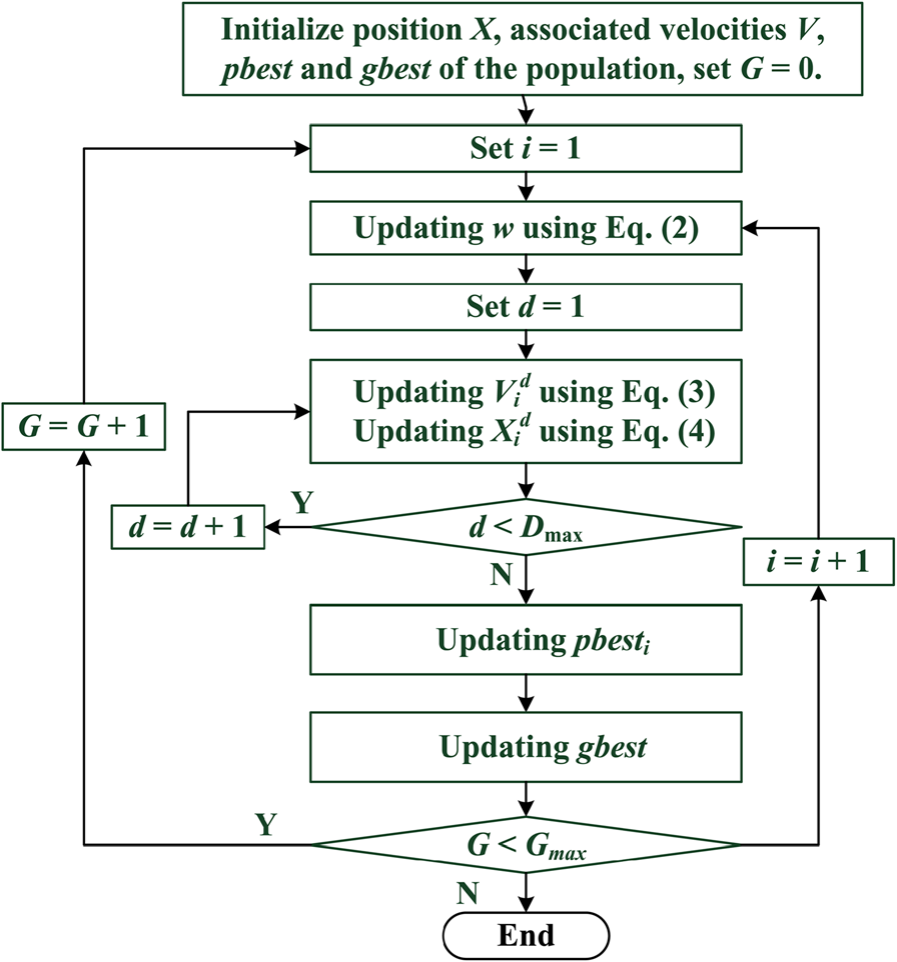
PSO flowchart.

Figure 1 shows the steps of the PSO procedure: (1) initializing the particles, (2) using an objective function to evaluate the particles, (3) selecting the particles' *pbest* and *gbest*, and (4) updating the velocity and position of the particles. These procedures are repeated in successive iterations until the termination conditions are reached. The elements of the proposed PSO include (1) particle, (2) population, (3) particle velocity, (4) particle position, (5) inertia weight, (6) individual best value, (7) global best value, and (8) termination criteria. These elements are defined as follows.

1. Particle: a problem solution consisting of several parameters.

2. Population: the set of all particles.

3. Particle velocity (*v*): each particle has a velocity, and the velocity of the *i*th particle is *v*_*i*_ = (*v*_*i*1_, *v*_*i*2_, …, *v*_*iD*_), where *v*_*iD*_ is the velocity of the *i*th particle with respect to the *D*th parameter.

4. Particle position (*x*): a vector consisting of available parameters in the practicable solution. The position of the *i*th particle is *x*_*i*_ = (*x*_*i*1_, *x*_*i*2_, …, *x*_*iD*_), where *x*_*iD*_ is the position of the *i*th particle with respect to the *D*th parameter. Each parameter in the particle vector is defined by the number of selected SNPs and their corresponding genotypes for the associated SNPs.

5. Inertia weight (*w*): the current velocity may be affected by the inertia weight, which determines whether the previous velocity increases/decreases the current velocity. Control factor *w* affects the trade-off between the exploration and exploitation capabilities of the particle.

6. Individual best fitness value (*pbest*)*:* the position of the *i*th particle with the highest fitness value (*pbest*_*i*_) is considered the best current solution for the *i*th particle.

7. Global best fitness value (*gbest*): the best particle in the population.

8. Termination criteria: the process stops when the maximum allowed number of iterations is reached.

### Encoding schemes

A vector of a particle is defined as the number of selected SNPs and their corresponding genotypes; SNPs cannot be repeatedly selected. Particle encoding can be represented as a vector as follows:

$$\begin{array}{l} {\mathrm{Particle}}_{i}=\left({\mathrm{SNP}}_{1},{\mathrm{SNP}}_{2},\ldots ,{\mathrm{SNP}}_{m},{\mathrm{Geno}}_{1,}{\mathrm{Geno}}_{2},\ldots ,{\mathrm{Geno}}_{m}\right),i\\ \;=1,2,\ldots ,n, \end{array}$$

where SNP represents the selected SNPs, Geno represents the SNP-corresponding genotypes (AA, Aa, and aa), and *n* represents the population size.

### Population initialization

The above encoding schemes are used for randomly generating the particle population over the problem space. SNPs are selected based on a randomly generated value among available SNPs. Three genotypes are also randomly generated between 1 and 3 to represent ‘AA’, ‘Aa’, and ‘aa’. The SNPs selected between SNP_1_ and SNP_*m*_ differ for each particle. If a given SNP is found to be repeated in a particle, the SNP is randomly generated among the available SNPs until it is different. For example, for a two-SNP particle with vector = (1, 4, 2, 3), SNPs 1 and 4 and genotypes 2 and 3 are chosen. The SNP barcodes (i.e., the SNPs and their corresponding genotypes) are represented as (1, 2) and (4, 3).

### Objective function

An objective function was used to compute the SNP barcode for the difference between the groups with high and low FEP values; the objective function was also used to compute the fitness value. The largest difference between the high and low groups was the highest fitness value. The equation was defined as follows:

(1) $$F\left(X_{i}\right)=\left(\mathrm{High}-\mathrm{FEP}\;\mathrm{group}\cap X_{i}\right)-\left(\mathrm{Low}-\mathrm{FEP}\;\mathrm{group}\cap X_{i}\right),$$

where ‘high-FEP group’ and ‘low-FEP group’ are the total number of SNP interactions in the high-FEP group and the low-FEP group, respectively. The *X*_*i*_ represents the *i*th particle. The high-FEP group ∩ *X*_*i*_ was defined as the total number of intersections between the high-FEP group and the *i*th particle. The low-FEP group ∩ *X*_*i*_ was defined as the total number of intersections between the low-FEP group and the *i*th particle. For example, *X =* (1, 2, 3, 1), i.e., SNP 1 with ‘aa’ and SNP 2 with ‘AA’, was used to evaluate the number of matching conditions in the two groups. The high-FEP group contained 30 samples, and the low-FEP group had 18 samples. According to Equation 1, the value was calculated by subtracting 18 from 30, leaving 12.

### Updating *pbest* and *gbest*

The personal best position (*pbest*) and the global best position (*gbest*) must be recorded for moving particles. If the current particle value is better than *pbest*, then the position and fitness value of *pbest* are updated with the current position and fitness value. If the fitness value of *pbest* is better than that of *gbest*, *gbest* is reset to *pbest*.

### Updating particle velocity and position

The search direction of a particle is determined by the three different vectors, i.e., *w*, *pbest*, and *gbest*. Equation 2 shows the *w* updating function, which iteratively reduces the value of *w* from *w*_max_ to *w*_min_, i.e., the function is a positive linear function of time that changes with the generations. The PSO algorithm applies Equation 3 to update the particle velocity. Equation 4 is the change in position (i.e., possible solution) needed to search for a better solution.

(2) $$\begin{array}{l} w_{\mathrm{LDW}}=\left(w_{\max}-w_{\min}\right)\times \frac{{\mathrm{Iteration}}_{\max}-{\mathrm{Iteration}}_{i}}{{\mathrm{Iteration}}_{\max}}\\ \;+w_{\min} \end{array}$$

(3) $$\begin{array}{l} v_{\mathrm{id}}^{\mathrm{new}}=w_{\mathrm{LDW}}\times v_{\mathrm{id}}^{\mathrm{old}}+c\times r_{1}\times \left(\mathrm{pbes}t_{\mathrm{id}}-x_{\mathrm{id}}^{\mathrm{old}}\right)\\ \;+c\times r_{2}\times \left(\mathrm{gbes}t_{d}-x_{\mathrm{id}}^{\mathrm{old}}\right) \end{array}$$

(4) $$x_{\mathrm{id}}^{\mathrm{new}}=x_{\mathrm{id}}^{\mathrm{old}}+v_{\mathrm{id}}^{\mathrm{new}},$$

where *w*_LDW_ is the inertia weight, *w*_max_ is 0.9, *w*_min_ is 0.4, and Iteration_max_ is the maximum number of allowed iterations. The *r*_1_ and *r*_2_, which are the random functions in the range [0, 1], are used to adjust the strength of the *pbest* and *gbest* vectors in a single generation. The *c*_1_ and *c*_2_ are acceleration constants that control the particle search direction (*pbest* or *gbest*). Velocities $v_{\mathrm{id}}^{\mathrm{new}}$ and $v_{\mathrm{id}}^{\mathrm{old}}$ represent the new and old velocities, respectively. The $x_{\mathrm{id}}^{\mathrm{old}}$ and $x_{\mathrm{id}}^{\mathrm{new}}$ are the current and updated particle positions, respectively. The velocity indicates the change required for a particle to obtain its global best position at a given moment in time, i.e., the velocity of the particle flying toward the best position.

### PSO parameter settings

As suggested by [34], the PSO parameters were optimized as follows. The population size was set to 50. The PSO termination condition was set to a pre-specified number of iterations (here, 100). The starting value of the inertia weight *w* was set to 0.9, and the final value was set to 0.4 [35]. Acceleration (learning) factors *c*_1_ and *c*_2_ were both set to 2 [36].

### Statistical analysis

Statistical analysis was performed using SPSS version 19.0 (SPSS Inc., Chicago, IL, USA). The OR with 95% confidence interval (CI) was used for performance measurements of the best SNP barcode; a *P* value smaller than 0.05 was considered a statistically significant difference between the two groups. Finally, G*Power 3 (Heinrich-Heine-Universität Düsseldorf, Germany) [37] was used to estimate the power.

## Results

### Example

The dataset used in this study was obtained from our previous study of associations with FEP [12] in a Taiwan population of 182 unrelated healthy subjects (82 men and 100 women). The Mayer-Salovey-Caruso Emotional Intelligence Test (MSCEIT) [38] was used to measure FEP in terms of perceiving, facilitating, understanding, and managing emotions [39]. The complete genotype data set is available at http://bioinfo.kmu.edu.tw/All_genotypes_for_low_high_FEP.xlsx.

The objective of this study was to evaluate the use of PSO for identifying the best combinations of SNPs in specific genotypes. The subjects had an average FEP score of 90.66 ± 1.49 (mean ± SE). Based on their scores, the subjects were dichotomized into a high-FEP group (FEP score > 90.66) and a low-FEP group (FEP score ≤ 90.66). Accordingly, PSO-generating SNP barcodes were coupled with the phenotype (FEP) to analyze 89 subjects with high FEP scores and 93 subjects with low FEP scores.

### Comparison of high- and low-FEP groups in terms of effects of single SNP

Table 1 shows the performance (*P* value) of each of five SNPs of two related genes (MET and AKT1). The occurrence of the genotype for each SNP did not significantly differ between the high- and low-FEP groups (*P* > 0.05).

**Table 1:** Single SNPs in high-FEP and low-FEP groups

**SNP no.**	**SNPs (genes)**	**Genotypes**	**High-FEP group ( *n *)**	**Low-FEP group ( *n *)**	***P *** **value**
1	rs1130233 (AKT1)	1-AA	22	33	
		2-GG	21	15	0.087
		3-AG	50	41	0.080
2	rs1858830 (MET)	1-GG	42	37	
		2-GC	44	42	0.797
		3-CC	7	10	0.370
3	rs2237717 (MET)	1-CC	25	28	
		2-CT	18	23	0.753
		3-TT	50	38	0.266
4	rs41735 (MET)	1-GG	25	29	
		2-AA	17	19	0.931
		3-GA	51	41	0.286
5	rs42336 (MET)	1-GG	17	20	
		2-AA	24	28	0.985
		3-GA	52	41	0.304

### The best SNP barcodes for the high- and low-FEP groups

Table 2 shows ten representative barcodes for combinations of two SNPs (two-SNP barcodes), and the magnitude of difference between the high- and low-FEP groups is presented from maximal to minimal. Out of all the combinations, the two-SNP barcodes, e.g., SNPs (1, 4) in genotype 3-3, [rs1130233-AG]-[rs41735-GA], showed the largest difference (12) between the high-FEP and low-FEP groups (30 vs. 18, respectively). The other two-SNP barcodes are provided on-line (http://bioinfo.kmu.edu.tw/All_2SNP_barcodes_for_FEP_scores.xlsx) along with all possible combinations of two SNPs with genotypes. The PSO was also used to obtain the SNP barcodes for the best-performing combinations of three to five SNPs (i.e., the combinations that obtained the largest differences between the high-FEP group and the low-FEP group).

**Table 2:** Ten representative differences in the occurrence of SNP barcodes for two-SNP combinations

**Combined SNPs**^**a**^	**Genotypes**^**a**^	**High-FEP group ( *n *)**	**Low-FEP group ( *n *)**	**Difference**^**b**^
SNPs(1,4)	3-3	30	18	12
SNPs(1,3)	3-3	27	17	10
SNPs(1,2)	3-1	27	18	9
SNPs(2,3)	2-3	28	21	7
SNPs(2,4)	2-3	28	22	6
SNPs(2,3)	1-2	13	8	5
SNPs(1,2)	2-2	8	4	4
SNPs(1,2)	2-1	11	8	3
SNPs(1,4)	2-1	6	4	2
SNPs(1,2)	3-3	3	2	1

^a^Numbers in parentheses indicates SNP combinations. For example, SNPs(1, 4) of genotypes 3-3 is the combination of [rs1130233-AG] and [rs41735-GA]. For detailed SNP data, see Table 1. ^b^Difference should be provided with footnote, i.e., difference = High-FEP group (*n*) − Low-FEP group (*n*).

### Associations between PSO-generated SNP barcodes and FEP

The left side of Table 3 shows the best PSO-generated SNP barcodes for combinations of two to five SNPs. The PSO algorithm identified the SNP interactions with the largest differences between the high- and low-FEP groups. For example, the largest difference in two-SNP interactions was in the SNP barcode for [rs1130233-AG] and [rs41735-GA]. The largest difference in three-SNP interactions was in the SNP barcode for SNPs 1, 4, and 5 of genotypes 3-3-3, i.e., [rs1130233-AG]-[rs41735-GA]-[rs42336-GA]. The largest difference in four-SNP interactions was in the SNP barcode for SNPs 1, 3, 4, and 5 of genotypes 3-3-3-3, i.e., [rs1130233-AG]-[rs2237717-TT]-[rs41735-GA]-[rs42336-GA]. Restated, the best SNP barcodes generated by PSO for fixed numbers of SNPs consistently obtained the largest differences in SNPs between the high- and low-FEP groups.

**Table 3:** The best SNP barcodes with the strongest associations with FEP performance

**SNP**^**a**^	**Genotypes**^**a**^	**High-FEP group**	**Low-FEP group**	**Difference**	***P *** **value**	**OR (95% CI)**^**c**^	**Power**
SNPs(1,4)	3-3	30	18	12	0.028	2.119 (1.077 to 4.168)	0.528
	Other^b^	59	75				
SNPs(1,4,5)	3-3-3	27	15	12	0.023	2.265 (1.109 to 4.624)	0.556
	Other^b^	62	78				
SNPs(1,3,4,5)	3-3-3-3	25	14	11	0.032	2.204 (1.059 to 4.586)	0.500
	Other^b^	64	79				
SNPs(1,2,3,4,5)	3-1-3-3-3	11	4	7	0.048	3.138 (0.960 to 10.253)	0.400
	Other^b^	78	89				

^a^For detailed SNP data, see Table 1. ^b^‘Other’ indicates reference group defined as the union of all other possible combinations of two to five SNPs. ^c^Difference should be provided with footnote, i.e., difference = High-FEP group (*n*) − Low-FEP group (*n*).

### The OR rankings indicate the SNP barcodes associated with high FEP

The right side of Table 3 shows the estimated effect (*P* value, OR, and 95% CI) of the best PSO-generated SNP barcodes in terms of FEP. The occurrence of the best SNP barcodes (combinations of two to five SNPs) significantly differed (*P* < 0.05) between the high-FEP group and the low-FEP group (range, 12 to 7 for barcodes with two to five SNPs). The OR values of the best SNP barcodes for two to five SNPs ranged from 2.119 to 3.138, the 95% CI of OR ranged from 1.059 to 10.253, and the power ranged from 0.556 to 0.400.

## Discussion

Single-SNP models are typically used to evaluate SNPs for associations with various diseases [40-44]. For some SNPs, however, effects that are potentially important but do not reach the significance level are underestimated, leading to the ‘missing heritability’ [45-47]. Recent psychiatry and neuroscience studies have investigated the importance of non-significant effects of SNPs. For example, studies of schizophrenia have revealed synergistic SNP-SNP interactions in dopamine receptor D4 (DRD4) [48] and in neuregulin 1 (NRG1)/v-erb-a erythroblastic leukemia viral oncogene homolog 4 (avian) (ERBB4)/AKT1 genes [49-51].

Our previous study [12] compared FEP scores among five SNPs of three genotypes in each of the MET and AKT1 genes. Chi-square test showed that the FEP scores significantly differed (*P* < 0.05) in only one genotype (rs2237717) out of three MET genotypes. However, the genotypes were not analyzed in terms of associations with FEP scores or in terms of all potential SNP-SNP interactions.

In the present study, the comparison of all SNPs in the MET and AKT1 genes between the high-FEP group and the low-FEP group showed no significant differences in terms of genotype frequency (Table 1). In contrast, the SNP barcodes generated by the proposed PSO algorithm for two to five SNPs showed significantly increased OR values (*P* < 0.05) (Table 3). This suggests that gene-gene interactions involving the MET and AKT1 genes may play an important role in FEP performance. The importance of this interaction is also partly supported by evidence that MET activates AKT1 phosphorylation in an anti-apoptotic pathway [52].

By comparing the maximum difference between the high-FEP and low-FEP groups for each SNP barcode, PSO can be used to determine the relative contribution of an SNP in FEP performance. For example, the OR values between the high- and low-FEP groups for two-SNP barcodes was 2.119. Adding SNP 5 or SNP 5/SNP 3 for a three-SNP barcode and a four-SNP barcode slightly increased the OR values between the high- and low-FEP groups, which suggests that SNP 1 and SNP 4 have larger effects on FEP performance compared to SNP 5 and SNP 3. Similarly, compared to SNP2, SNP 5, and SNP 3 had larger effects on high-FEP performance. Accordingly, the relative strengths of the effects of individual SNPs on FEP performance are apparently as follows: SNP 1 (rs1130233)/SNP 4 (rs41735) > SNP 5 (rs42336)/SNP 3 (rs2237717) > SNP 2 (rs1858830).

Although this study applied the PSO algorithm in a small sample size of SNPs, the algorithm has proven effective for large datasets [53,54] such as genome-wide association studies [30,31]. Therefore, this PSO methodology is applicable for further study of SNP-SNP interactions not only in psychiatry but also in other research fields. Additionally, with the help of power calculation, the detectable minimum effect size can be predicted for suitable sample collection to improve the performance of PSO computation.

In conclusion, FEP is an essential component in social cognition and social skill not only in healthy individuals but also in patients with mental disorders such as schizophrenia [1-4]. This study confirmed that the proposed PSO algorithm can identify SNP barcodes for high-FEP performance in a healthy Taiwan population. Although further studies are needed to determine whether the findings can be extrapolated to mental disorders or to other racial populations, the FEP impact for SNPs of missing heritance was sensitively detected in this SNP-SNP interaction mode in the current study.

In conclusion, the contribution of this study is a computational methodology for analyzing complex SNP-SNP interactions in social cognition domains such as FEP performance. Although each SNP has a different impact on FEP, improved understanding of the joint effects of SNP-SNP interactions further elucidates the molecular mechanisms of facial emotion identification.

## Competing interests

The authors declare that they have no competing interests.

## Authors' contributions

CHY, and HWC wrote the manuscript. HYL and MTL designed the study. CHY, LYC, and YDL performed computation and conducted the statistical analysis. CHY and HWC interpreted the data. All authors read and approved the final manuscript.
